# Associations between Psychosocial Working Conditions and Work-Specific Self-Efficacy Beliefs Among Employees Receiving Psychotherapeutic Consultation at Work

**DOI:** 10.1007/s10926-024-10256-1

**Published:** 2024-11-22

**Authors:** Jeannette Weber, Marieke Hansmann, Meike Heming, Regina Herold, Yesim Erim, Nicole Hander, Eva Rothermund, Nadine Mulfinger, Christoph Kröger, Manuel Feißt, Jolanda Brezinski, Fiona Kohl, Peter Angerer

**Affiliations:** 1 Institute of Occupational, Social and Environmental Medicine, Centre for Health and Society, Medical Faculty and University Hospital Düsseldorf, Heinrich Heine University Düsseldorf, Moorenstraße 5, 40225 Düsseldorf, Germany; 2 Institute of Psychology, University of Hildesheim, Hildesheim, Germany; 3 Department of Psychosomatic Medicine and Psychotherapy, University Hospital of Erlangen, Friedrich-Alexander University Erlangen-Nürnberg (FAU), Erlangen, Germany; 4 Department of Psychosomatic Medicine and Psychotherapy, Ulm University Medical Center, Ulm, Germany; 5 Department of Psychiatry II, Ulm University and BKH Günzburg, Günzburg, Germany; 6 Institute of Medical Biometry, University of Heidelberg, Heidelberg, Germany

**Keywords:** Occupational stress, Workplace, Mental health, Occupational health, Psychotherapeutic care, Self-efficacy

## Abstract

**Purpose:**

By considering work-related aspects during early intervention and treatment of employees with (subclinical) symptoms of common mental disorders, **p**sycho**t**herapeutic consultation at **w**ork (PT-W) aims to increase work-specific self-efficacy (SE) to finally reduce sickness absence and contribute to successful return to work. This study, thus, aims to investigate interrelations between working conditions and work-specific SE among employees before receiving PT-W.

**Methods:**

The study uses baseline data of a randomized controlled trial testing the efficacy of PT-W in Germany (n = 535). Working conditions were assessed by six scales of the validated Copenhagen Psychosocial Questionnaire (COPSOQ). SE was assessed by the validated general short occupational self-efficacy (OSE) scale and return-to-work self-efficacy (RTW-SE) scale, two specific forms of self-efficacy. Multiple linear regression models were calculated using working conditions as independent and self-efficacy as dependent variables. Interactions between working conditions and age, gender and current extent of work were added to those models.

**Results:**

Results suggest that quantitative job demands are negatively and development opportunities are positively related to OSE and RTW-SE. Age did not moderate those relationships. The association between development opportunities and OSE was stronger among employees indicating working less number of hours than specified in their contract compared to employees indicating working their full contract hours. Furthermore, interactions with gender were found with social support being only (positively) associated with OSE among male and decision authority being only (positively) associated with OSE among female employees.

**Conclusions:**

The associations between working conditions and work-specific SE support the usefulness of addressing potential reciprocal relationships between those two variables during PT-W to improve mental health of employees.

**Trial registration number:** Registered at the German Clinical Trial Register (DRKS) at 01.03.2021—DRKS00023049.

**Supplementary Information:**

The online version contains supplementary material available at 10.1007/s10926-024-10256-1.

## Introduction

Meta-analytical findings showed that work-specific self-efficacy beliefs (SE) are an important predictor for return to work among employees with common mental disorders (CMD; [1]) and working conditions are discussed to influence work-specific SE [2–6]. By providing first access to psychotherapeutic care to employees with mental health problems [7, 8], psychotherapeutic consultation at work (PT-W) may address working conditions as well as work-specific SE during work-focused treatment [9–13]. To give some examples, this could include fostering work-specific SE or elaboration of strategies to cope with adverse working conditions [10, 12, 13]. Studying interrelationships between those two factors among employees before receiving PT-W is, therefore, highly important to inform therapists which working conditions might be useful to address during PT-W to improve work-specific SE.

SE describe individual’s beliefs in his or her ability to successfully perform a particular behaviour [14]. SE influence the goals people set and the persistence of goal striving in the face of challenges [15]. There are three forms of SE that differ in regard to their generality: General, domain-specific and task-specific SE [16, 17]. While general SE refer to the perceived ability to perform across a variety of situations and settings, domain-specific SE relate to a specific area or setting, e.g. family, school or work [18, 19], whereas task-specific SE refers to single tasks that may differ during work [20]. It has been discussed that measures of task-specific self-efficacy are more valid when the task is clearly defined and somewhat familiar to individuals [21]. With regard to the occupational domain, empirical studies predominantly investigated general occupational SE (OSE, [22]) or return-to-work SE (RTW-SE, e.g. [23–25]). Both, OSE and RTW-SE, refer to the confidence to fulfil different job tasks successfully [22, 25]. However, RTW-SE beliefs can be considered slightly more specific as they represent the confidence in one’s coping ability despite an existing mental illness and with respect to disability-specific challenges that are relevant during and after RTW for employees with mental health problems (e.g. difficulty in concentrating, problems with energy regulation [25]). In earlier studies, OSE was positively associated with job satisfaction and job performance and negatively associated with perceived job insecurity (e.g. [22, 26]). Moreover, meta-analytical results show that higher levels of RTW-SE are a strong prognostic factor for better RTW outcomes in sick-listed employees with mental disorders [1].

It is conceivable that working conditions might affect perceptions of OSE and RTW-SE. According to the job-demand-resources model (JD-R) [27], job demands may be conceived as factors that might jeopardize well-being, lead to burnout and decrease motivation in the context of work whereas decision authority, development opportunities, leadership quality and social support may act as resources [28]. High demands in combination with high resources may protect mental health and increase motivation and work engagement, respectively. Motivation and work engagement in turn are closely related to SE and SE can act as a mediator between working conditions and health or work engagement [29–31].

In that respect, unfavourable working conditions such as high job demands might diminish beliefs to be able to accomplish one’s work [6]. Conversely, job resources including high decision authority, development opportunities, leadership quality or social support might activate SE by providing employees with important means to control their job and achieve their work goals [2]. Furthermore, working conditions were shown to be important predictors for actual RTW [32], and therefore, associations between working conditions and RTW-SE seem likely. Cross-sectional studies confirmed that working conditions (e.g. high work pace and workload [5] and job stress (e.g. high demands, low control and low support [3, 4]) are associated with OSE among non-clinical study populations [3, 4] and with RTW-SE among employees with CMD being on sick leave [5]. In addition, also a longitudinal study investigating employees working in private sector organizations provided evidence that role conflict, as a type of job demand, shapes OSE [6].

In addition to direct associations, also individual or job characteristics could affect the association between the working conditions above mentioned and SE. First, current extent of work might be a moderator, suggesting that associations might vary depending on whether employees are still on the job or sick listed. Second, the meaning and impact of various working conditions might change during lifetime [33]. For example, it was suggested that older employees are more likely to efficiently work with high job control due to their work experience, whereas younger employees would need more guidance and, thus, less control [34]. This could lead to a stronger association between job control and SE among older compared to younger employees. Third, gender might also moderate associations between working conditions and SE. For example, previous studies suggest that development opportunities might be stronger predictors for work ability among male employees, whereas leadership quality and decision authority seemed to be more important among female employees [35, 36].

Psychotherapeutic consultation at work (PT-W) offers employees first and low threshold access to psychotherapeutic care when experiencing mental distress or other symptoms of CMD [7, 8]. It aims to avoid times of sickness absence by improving symptoms, prognosis and reducing the risk of chronification of mental disorders [8, 37]. Most PT-W concepts include a needs assessment, diagnosis and recommendations for further treatment in care as usual [11, 38, 39]. A current concept of PT-W also incorporates collaboration between psychotherapists and occupational health professionals as well as work-focused treatment [11] targeting symptom reduction, improvement of work ability and restoration and improvement of work-specific SE [9, 10, 12, 13]. PT-W therapists may, thus, address working conditions as well as work-specific SE during work-focused treatment [9–13]. Investigating the association between working conditions and work-specific SE among employees before receiving PT-W, therefore, seems to be a research gap needing attention. Considering RTW-SE as well as OSE in this context might be relevant. On the one hand, PT-W offers secondary prevention for employees with early symptoms of CMD [8, 11], and therefore, the more general concept of OSE seems appropriate to use in this population. On the other hand, PT-W may also include tertiary prevention offers for employees with a manifest diagnosis of CMD planning to return to work after sick leave [11]. The concept of PT-W aims at enhancing work-specific SE (OSE and RTW-SE) as a main focus. It is, therefore, important to study associations between working conditions and OSE as well as RTW-SE among employees before receiving PT-W. This study will, thus, explore which working conditions are associated with OSE and RTW-SE among employees with (subclinical) symptoms of CMD before receiving PT-W. It will further explore whether age, gender and current extent of working moderate those associations.

We hypothesize that (1) work demands are negatively related to SE and (2) that decision authority, development opportunities, leadership quality and social support are positively related to SE.

## Methods

### Study Design

Baseline data of a multicentre randomized controlled trial (friaa-study) was used [11]. The friaa-study investigates whether PT-W is superior in reducing days of sickness absence compared to treatment as usual. For this purpose, PT-W was established for various small, middle and large-sized companies around five study centres in Germany. Participants were recruited through various channels and disseminators including occupational health services and social counselling, human resources departments, supervisors, company intranet, newsletters, mailing lists and flyers. Furthermore, employees were also recruited by social media campaigns in some study centres and then participated in the study independent from their company affiliation. Each participant filled out an online baseline questionnaire and then received a basic clinical diagnostic assessment within a first session of PT-W. Within this session, employees were also screened for their eligibility for the study. All participants were then randomized into a control or intervention group by 1:1 allocation. The control group received recommendations regarding standard care and a follow-up phone call to offer further support, whereas the intervention group received one session of a work-related assessment and up to 15 further treatment sessions. All participants gave their written informed consent to the study. The study was approved by the ethics committees of all study centres (Ulm University: Application No. 339/20; Friedrich-Alexander University Erlangen-Nürnberg: Application No. 525_20 Bc; University of Hildesheim: Application No. 165; Heinrich-Heine-University Düsseldorf: Application No. 2021–1279). A detailed description of the friaa-study is given elsewhere [11].

### Study Participants

This cross-sectional analysis includes baseline data of the control and intervention group. Since the baseline questionnaire was filled out before randomization, no group differentiation is considered in this study. Eligibility criteria followed the study protocol [11]. Inclusion criteria were (i) aged ≥ 18 years, (ii) sufficient command of German to participate in the study, (iii) weekly working hours ≥ 15 h, (iv) diagnosis of CMD or symptoms of psychosomatic disorders. Exclusion criteria were (i) main diagnosis of substance abuse, schizophrenia, psychosis, organic psychiatric disorders, (ii) severe somatic health problems, (iii) current use of psychotherapeutic care and (iv) application for retirement pension. Participants indicating diverse gender were additionally excluded for this analysis due to their small number in the study (*n* = 1).

### Measures

Psychosocial working conditions: Psychosocial working conditions were measured by the third German version of the validated Copenhagen Psychosocial Questionnaire (COPSOQ; [40]). The following sub-scales were used: quantitative job demands (five items), emotional demands (two items), decision authority (two items), development opportunities (three items), leadership quality (four items) and social support (four items). Example items are “Do you have to work very fast?” for quantitative job demands and “Do you have the possibility of learning new things through your work?” for development opportunities [40, 41]. All items were either answered on a five-point rating scale from 100 = “always” to 0 = “never or hardly never” or from 100 = “to a very large extent” to 0 = “to a very small extent”. Mean scores were calculated for each sub-scale with higher values indicating more quantitative and emotional job demands, decision authority, development opportunities, leadership quality and social support. Reliability was good for emotional demands [Cronbach’s alpha (CA) = 0.84], leadership quality (CA = 0.86) and social support (CA = 0.80), acceptable for development opportunities (CA = 0.76) and quantitative job demands (CA = 0.79) and questionable for decision authority (CA = 0.61).

General occupational self-efficacy: OSE was measured by the German version of the validated short occupational self-efficacy scale [22]. This scale consists of six items with a six-point rating scale from 1 = “not at all true” to 6 = “completely true”. An example item is “I can remain calm when facing difficulties in my job because I can rely on my abilities” [22]. A mean score was calculated with higher values indicating higher OSE. Reliability was good (CA = 0.87).

Self-efficacy regarding return to work: RTW-SE was measured by the validated German version of the return-to-work self-efficacy scale [24, 25]. This scale consists of eleven items asking participants on what they expect if they resumed their work fully on the following day. Each item has a six-point rating scale from 1 = “totally disagree” to 6 = “totally agree”. An example item is “I will be able to perform my tasks at work” [25]. Inverse items were recoded and a mean score was calculated with higher values indicating higher RTW-SE. Reliability was good (CA = 0.88).

Covariables: Participants were also asked about their gender (male, female, diverse), age, weekly working hours and to what extent they are currently working their contract hours (completely, reduced or not at all). Participants further indicated their occupational group with following options: unskilled blue-collar worker, skilled blue-collar worker, foremen/master craftsmen, ordinary employee, mid-level employee, executive employee, ordinary civil servant, mid-level civil servant, executive civil servant, self-employed, other. For the analyses, those options were further categorized as blue and white-collar workers. Blue-collar workers included unskilled or skilled blue-collar workers as well as foremen/master craftsmen. White-collar workers included ordinary, mid-level or executive employees and civil servants as well as self-employed participants. Participants indicating “other” were asked for a further description of their work and were then manually categorized into white or blue-collar workers.

### Statistical Analyses

All analyses were performed with R Version 4.2.2. Only complete cases were used for the analyses. Correlation analyses were performed between working conditions, OSE and RTW-SE using Pearson correlation. Multiple linear regression analyses were conducted with quantitative job demands, emotional demands, decision authority, development opportunities, leadership quality and social support as independent variables and OSE or RTW-SE as the dependent variable. All analyses further included the variables gender, age and current extent of work as well as weekly working hours and occupational group. In a first step, only direct relationships between those variables were calculated. In a second step, also interactions between psychosocial working conditions and either sex, age or current extent of work were included to the regression models. Package sjPlot with function plot_model in R was used to visualize interaction effects based on those regression models [42]. Furthermore, separate models of the first step were calculated for male and female participants as well as for the three manifestations of current extent of working (i.e. completely, reduced, not at all) if significant interactions occurred. Due to the interaction analyses, all continuous variables including OSE, RTW-SE, psychosocial working conditions, working hours and age were z-standardized prior to analyses. Multicollinearity was tested on the models without interaction terms using the variance inflating factor (VIF). Statistical significance is assumed at a p-level of < 0.05. Due to the reduced power of interaction analyses, interaction with p-levels between 0.05 and < 0.10 are reported as a trend and their interaction plots are shown in supplemental material.

## Results

There were 550 employees participating within the study. Fourteen participants were excluded from the analyses due to missing data and one participant due to the indication of diverse gender. Description of the remaining 535 participants is given in Table 1. Correlations between study variables are shown in Table 2.

**Table 1:** Description of the study sample (*n* = 535)

	Number (frequency in %)
*Sex*	
Female	294 (55.0%)
Male	241 (45.0%)
*Occupational group*	
Blue-collar worker	127 (23.9%)
White-collar worker	408 (76.1%)
*Current extent of work*	
Completely	412 (77.0%)
Reduced	54 (10.1%)
Not at all	69 (12.9%)

	Mean (standard deviation)	Minimum—Maximum
Age	46.01 (10.97)	21–64
Weekly working hours	36.83 (7.15)	7–75
*Psychosocial working conditions*		
Quantitative demands	55.65 (21.08)	0–100
Emotional demands	48.79 (32.45)	0–100
Decision authority	39.72 (22.69)	0–100
Development opportunities	63.41 (21.59)	0–100
Leadership quality	39.85 (25.20)	0–100
Social support	59.24 (22.53)	0–100
General occupational self-efficacy	3.88 (0.99)	1–6
Self-efficacy regarding return to work	3.52 (0.90)	1–6

**Table 2:** Pearson correlation between study variables

	1	2	3	4	5	6	7	8
1 Quantitative demands	1.00							
2 Emotional demands	0.29***	1.00						
3 Decision authority	0.00	0.10*	1.00					
4 Development opportunities	0.26***	0.21***	0.44***	1.00				
5 Leadership quality	− 0.15***	− 0.06	0.37***	0.30***	1.00			
6 Social support	− 0.15***	− 0.08	0.29***	0.25***	0.66***	1.00		
7 OSE	− 0.05	0.02	0.19***	0.29***	0.14***	0.18***	1.00	
8 RTW-SE	− 0.13**	0.00	0.12**	0.20***	0.19***	0.21***	0.60***	1.00

*OSE* general occupational self-efficacy, *RTW-SE* self-efficacy regarding return to work; *p* values: * < 0.05, ** < 0.01, *** < 0.001

### Results on General Occupational Self-Efficacy

Results of regression analyses predicting OSE are shown in Table 3. Direct associations were found with quantitative job demands and development opportunities. OSE was negatively associated with quantitative job demands and positively associated with development opportunities, meaning that participants who either indicated lower quantitative demands or higher development opportunities experienced higher levels of OSE. No direct associations were found with emotional job demands, decision authority, leadership quality and social support. The VIFs ranged between 1.04 and 1.92, suggesting that there is no multicollinearity problem in the model.

**Table 3:** Regression analyses predicting general occupational self-efficacy

	Model 1: without interactions		Model 2: Interactions with age		Model 3: Interactions with gender		Model 4 Interactions with current extent of work	
	B	SE	B	SE	B	SE	B	SE
Age	0.056	0.043	0.046	0.044	0.069	0.043	0.055	0.043
Gender (female vs. male)	0.121	0.089	0.119	0.090	0.128	0.089	0.081	0.090
Working hours	0.107*	0.043	0.105*	0.044	0.105*	0.043	0.106*	0.044
Occupation (white vs. blue collar)	− 0.070	0.104	− 0.074	0.105	− 0.085	0.104	− 0.085	0.104
Current extent of work (reduced vs. completely)	− 0.077	0.138	− 0.084	0.139	− 0.049	0.137	− 0.072	0.144
Current extent of work (not at all vs. completely)	− 0.274*	0.126	− 0.263*	0.123	− 0.264*	0.125	− 0.339*	0.136
Quantitative job demands	− 0.119*	0.048	− 0.119*	0.048	− 0.079	0.071	− 0.153**	0.054
Emotional job demands	− 0.022	0.044	− 0.022	0.044	− 0.076	0.070	− 0.011	0.048
Decision authority	0.055	0.049	0.059	0.050	− 0.051	0.069	0.079	0.055
Development opportunities	0.288***	0.050	0.289***	0.050	0.386***	0.075	0.236***	0.054
Leadership quality	− 0.049	0.057	− 0.048	0.057	− 0.112	0.084	− 0.012	0.063
Social support	0.105	0.056	0.103	0.056	0.260**	0.085	0.072	0.062
*Interactions with …*			*…age*		*…gender (female vs. male)*		*…current extent of work (reduced vs. completely)*	
Quantitative job demands			− 0.029	0.050	− 0.083	0.093	0.065	0.157
Emotional job demands			0.006	0.044	0.114	0.090	− 0.149	0.174
Decision authority			− 0.024	0.051	0.234*	0.099	− 0.214	0.177
Development opportunities			− 0.006	0.050	− 0.195^+^	0.099	0.477*	0.231
Leadership quality			0.066	0.058	0.107	0.113	− 0.053	0.210
Social support			− 0.010	0.054	− 0.276*	0.111	− 0.143	0.230
							*…current extent of work (not at all vs. completely)*	
Quantitative job demands							0.188	0.142
Emotional job demands							0.001	0.139
Decision authority							0.004	0.158
Development opportunities							0.272	0.167
Leadership quality							− 0.235	0.179
Social support							0.251	0.157
Model fit	Adj. *R*^2^ = 0.111, *F* = 6.579 (12,522), *p* *<* 0.001		Adj. *R*^2^ = 0.105, *F* = 4.496 (18,516), *p* < 0.001		Adj. *R*^2^ = 0.130, *F* = 5.412 (18,516), *p* < 0.001		Adj. *R*^2^ = 0.119, *F* = 4.013 (24,510), *p* < .001	
Effect size	*F*^2^ = 0.151		*F*^2^ = 0.157		*F*^2^ = 0.189		*F*^2^ = 0.189	

*B* unstandardized regression coefficient, *SE* standard error; *p* values: ^+^ < 0.10, * < 0.05, ** < 0.01, *** < 0.001

**Fig. 1:**
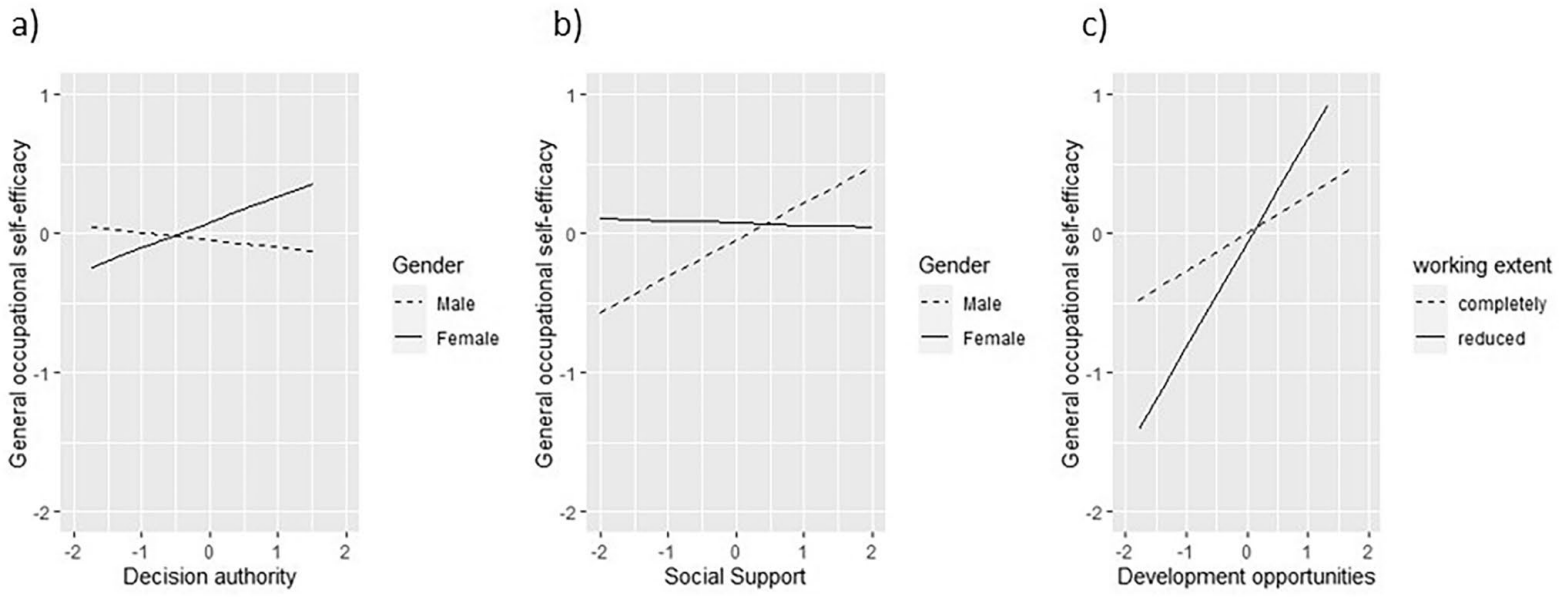
Interactions between decision authority and gender (a), social support and gender (b) and development opportunities and current extent of working (c) on general occupational self-efficacy

No interactions between working conditions and age were observed. However, moderator effects of gender on the association between OSE and decision authority and social support occurred. Decision authority was positively associated with OSE among female (*B* = 0.174, SE = 0.070, *p* = 0.01) but not among male participants (*B* = − 0.029, SE = 0.069, *p* = 0.67). Social support, in turn, was positively associated with OSE among male (*B* = 0.241, SE = 0.077, *p* = 0.003) but not among female participants (*B* = − 0.000, SE = 0.075, *p* = 1.00). Those interactions are graphical depicted in Fig. 1a, b. Furthermore, an interaction trend indicated that higher development opportunities are more strongly related with higher levels of OSE in male (*B* = 0.398, SE = 0.071, *p* < 0.001) than female (*B* = 0.180, SE = 0.070, *p* = 0.01) participants (Online Resource 1, Fig. S1). Regarding current extent of work, an interaction on the association between development opportunities and OSE was observed. Participants indicating reduced work showed a stronger positive relationship between development opportunities and general occupational self-efficacy (*B* = 0.613, SE = 0.178, *p* = 0.001) as compared to participants indicating that they were working their complete contract hours (*B* = 0.256, SE = 0.057, *p* < 0.001). This interaction is depicted in Fig. 1c. No other interactions between current extent of work and working conditions on OSE were observed.

### Results on Self-Efficacy Regarding Return to Work

Results of regression analyses predicting RTW-SE are shown in Table 4. Higher levels of RTW-SE were associated with lower levels of quantitative job demands and higher levels of development opportunities. Again, no associations were found with emotional job demands, decision authority, leadership quality and social support. The VIFs ranged between 1.040 and 1.930, which suggests that no multicollinearity problem exists for this model.

**Table 4:** Regression analyses predicting self-efficacy regarding return to work

	Model 1: without interactions		Model 2: Interactions with age		Model 3: Interactions with gender		Model 4 Interactions with current extent of work	
	B	SE	B	SE	B	SE	B	SE
Age	0.041	0.041	0.037	0.042	0.045	0.042	0.035	0.042
Gender (female vs. male)	0.027	0.085	0.022	0.086	0.023	0.086	− 0.003	0.087
Working hours	0.057	0.041	0.058	0.042	0.052	0.042	0.058	0.042
Occupation (white vs. blue collar)	− 0.171	0.099	− 0.175	0.099	− 0.184^+^	0.101	− 0.159	0.100
Current extent of work (reduced vs completely)	− 0.439***	0.132	− 0.424**	0.132	− 0.431**	0.132	− 0.477***	0.138
Current extent of work (not at all vs. completely)	− 0.985***	0.120	− 0.998***	0.121	− 0.975***	0.121	− 0.991***	0.130
Quantitative job demands	− 0.135**	0.046	− 0.142**	0.046	− 0.126^+^	0.069	− 0.117*	0.052
Emotional job demands	0.009	0.042	0.016	0.043	0.062	0.067	0.020	0.047
Decision authority	− 0.016	0.047	− 0.014	0.048	− 0.078	0.067	− 0.024	0.053
Development opportunities	0.220***	0.048	0.220***	0.048	0.247***	0.073	0.199***	0.052
Leadership quality	0.021	0.054	0.016	0.054	− 0.020	0.081	0.036	0.061
Social support	0.095	0.053	0.097^+^	0.053	0.205*	0.082	0.088	0.060
*Interactions with …*			*…age*		*…gender (female vs male)*		*…current extent of work (reduced vs. completely)*	
Quantitative job demands			0.091^+^	0.048	− 0.029	0.090	− 0.116	0.152
Emotional job demands			− 0.059	0.042	− 0.076	0.087	− 0.154	0.167
Decision authority			− 0.010	0.048	0.122	0.095	− 0.099	0.170
Development opportunities			− 0.040	0.048	− 0.053	0.097	0.333	0.223
Leadership quality			0.035	0.055	0.061	0.109	0.104	0.202
Social support			0.047	0.051	− 0.188^+^	0.107	− 0.269	0.221
							*…current extent of work (not at all vs. completely)*	
Quantitative job demands							0.008	0.134
Emotional job demands							− 0.024	0.134
Decision authority							0.159	0.152
Development opportunities							− 0.001	0.161
Leadership quality							− 0.257	0.172
Social support							0.226	0.152
Model fit	Adj. *R*^2^ = 0.189, *F* = 11.340 (12,522), *p* < 0.001		Adj. *R*^2^ = 0.190, *F* = 7.951 (18,516), *p* < 0.001		Adj. *R*^2^ = 0.188, *F* = 7.861 (18,516), *p* < 0.001		Adj. *R*^2^ = 0.184, *F* = 6.028 (24,510), *p* < 0.001	
Effect size	*F*^2^ = 0.261		*F*^2^ = 0.277		*F*^2^ = 0.274		*F*^2^ = 0.284	

*B* unstandardized regression coefficient, *SE* standard error; *p* values: ^+^ < 0.10, * < 0.05, ** < 0.01, *** < 0.001

A trend of an interaction occurred between age and quantitative job demands: the negative relationship between quantitative job demands and RTW-SE decreased in strength by increasing age (Online Resource 1, Figure S2). A trend of an interaction was further shown between social support and gender (Online Resources 1, Figure S3). Higher levels of social support were associated with higher levels of RTW-SE in male (B = 0.206, SE = 0.079, p = 0.01) but not female (B = 0.034, SE = 0.071, p = 0.63) participants. No other interactions between working conditions and age, gender or current extent of work emerged.

## Discussion

This study investigated associations between working conditions and work-specific SE in employees before they visited PT-W. Results suggest that higher levels of OSE and RTW-SE are associated with less quantitative job demands and more development opportunities. Gender-specific associations occurred regarding social support and decision authority. Higher levels of social support were only associated with higher OSE among male and higher levels of decision authority were only associated with higher OSE among female employees.

The finding that working conditions are associated with OSE and RTW-SE are in line with assumptions of the JD-R model [27, 28] as well as previous research. Those prior findings suggested that work pace, workload, authentic leadership, role conflicts and overall measures of working conditions and job stress are associated with work-specific SE [3–6]. Self-efficacy theory suggests that experiences of success and failure shape SE [14]. Our results may, thus, imply that lower levels of quantitative demands could be associated with increased work-specific SE by facilitating the experience to be able to accomplish the amount of work. By contrast, high quantitative demands could be associated with experiences of failure [43, 44] by not meeting all job demands successfully and, therefore, could diminish SE. Furthermore, the results might suggest that also development opportunities could be associated with SE, probably by giving employees opportunities to optimize, develop and refine skills that they need to fulfil their work tasks. However, SE might have also shaped perceptions of working conditions or triggered employees to proactively improve working conditions called job crafting [6, 45, 46]. For example, someone having a high confidence in one’s working abilities might evaluate the same amount of work as less demanding and threatening as someone with low confidence in one’s work [46]. Furthermore, reciprocal relationships have been described as positive gain spirals according to the Conservation of Resources (COR) theory [47] with SE playing a central, mediating role in the effects of job resources on well-being and vice versa [48, 49]. In other words, good working conditions could lead to increased work-specific SE, which then increase well-being and decrease the risk for mental illnesses such as depression. Higher well-being might in turn lead to increased work-specific SE, which finally leads employees to evaluate their working conditions as more positive or to work on unfavourable working conditions. Supporting evidence for reciprocal relationships between working conditions and well-being comes from a meta-analysis on longitudinal studies [50]. However, a previous longitudinal study that investigated whether OSE mediates the relationships between working conditions and well-being could not confirm reciprocal effects. This study provided evidence that role conflict, as a type of job demand, shapes OSE, but it gave no support for reverse causation (i.e. OSE predicting role conflict [6]).

In addition to quantitative demands and development opportunities, we also found gender-specific associations between work-specific SE and social support and decision authority. To the best of our knowledge, this has been the first study so far, which investigated gender-specific associations between working conditions and work-specific SE. Studies examining other outcomes have found inconsistent results regarding gender-specific effects of working conditions (e.g. [51–54]). Whereas a meta-analysis from 2006 found that social support at work is more strongly related to mental health in males [54], newer studies found stronger effects for women or similar effects for women and men [51–53]. However, one study investigating work ability as an outcome among older employees supports our results by finding a relationship with social support among male but not among female employees [35]. Those findings are in contrast to the social-role theory, which suggests that individuals’ beliefs, attitudes and behaviour is influenced by gender roles in their society [55]. Since Western societies rather connect female roles with caring and communion, one might have expected that social support is more important for women than men [56]. Why our study found contrasting results needs to be explored further. One explanation for the lack of association between social support and work-specific SE among female employees could include the possibility that women might have a wider supportive network outside work and, therefore, do not rely on social support at work as strongly as male employees do [54]. Furthermore, the importance of social support may change with respect to both, age and gender. However, our study sample is too small to test in a three-way interaction.

Our finding that decision authority and OSE are only associated with each other among female employees is supported by another previous study investigating work ability instead of OSE as an outcome in a population of the general workforce [36]. An explanation for this observation is lacking so far, but it might be conceivable that stronger responsibilities at home for female employees [57] could play a role. Previous research suggests that female employees may reduce their work-family conflict by using decision authority at work (e.g. control over working times and breaks) to juggle work and private responsibilities [58, 59]. Since work-family conflict and SE were repeatedly found to be associated with each other (e.g. [60, 61]), this could explain the relationship between OSE and decision authority among female participants. Compared to findings for OSE, no moderated or direct effects of decision authority on RTW-SE occurred. This result contrasts a previous research finding including a direct effect of job autonomy on RTW-SE among employees with mental disorders [62]. The main difference of the two studies lies in the study population, because the former study only included participants not being back to work due to their health condition [62]. However, we could also not find an interaction between decision authority and current extent of working on RTW-SE. Reasons for those disparate findings, thus, need to be explored further.

Age and current extent of work seemed to be less important for the relationship between working conditions and work-specific SE in our study population. One exception was the relationship between development opportunities and OSE, whereby higher levels of development opportunities were more strongly related with higher levels OSE among employees working reduced hours than among employees working their complete hours. Furthermore, the coefficients of determination were rather low for the regression models indicating that only 11 to 19% of the variance of the two work-specific SE scales were explained by our independent variables.

Generally, we found similar results for OSE and RTW-SE. Our findings, thus, suggest that working conditions might not only affect the slightly more general OSE but also RTW-SE, which specifically addresses person-centred disease-related aspects of SE [25]. The similar findings are not surprising, because OSE and RTW-SE were highly correlated with each other in this study. Also previous research found that RTW-SE is strongly related with more general measures of SE [25]. However, correlation between OSE and RTW-SE was not perfect and, therefore, suggests that RTW-SE is a unique construct [25]. The result that current extent of work was especially and more clearly associated with RTW-SE than with OSE further supports construct validity of RTW-SE, because it was especially developed as a SE-scale for employees being absent from work due to illness [23].

### Limitations

This study has several limitations. First, the cross-sectional analysis might not infer causality or gives indications for the direction of relationships. Nonetheless, the results might guide longitudinal studies regarding which working conditions might especially be useful to explore further and that gender-sensitive analyses are advisable.

Second, a common method bias cannot be excluded, because only self-reported questionnaire data were used. This could have led to an underestimation of interaction effects [63]. However, we also reported interaction trends that missed statistical significance to compensate for reduced power in interaction analyses. Furthermore, a five-point Likert scale was used to assess working conditions, whereas a six-point Likert scale was used for SE reducing the risk of common method bias. In addition, the use of multiple linear regression in this study with a large number of independent variables likely further reduces the risk of common method bias [63].

Third, the study only included employees before having received PT-W, which is a limitation and strength at the same time. On the one hand, especially employees might have participated who experienced interrelations between adverse working conditions and mental health due to the work focus of PT-W. Therefore, the results might not be generalizable to employees who do not visit PT-W. On the other hand, the result that working conditions are associated with work-specific SE in this study group supports the concept of PT-W, which includes a work-related assessment and work-focused treatment with the aim to increase work-specific SE [11].

Fourth, internal consistency of the decision authority scale was questionable, which might limit the informative value of associations between decision authority and SE. However, this low internal consistency might be explained by the low number of items of the decision authority scale, because internal consistency generally increases with the number of items [64].

### Implications

This study gives first evidence that it might be useful to address working conditions and work-specific SE as well as to detect their interrelations with each other during PT-W with the objective to increase work-specific SE. This may help support successful RTW, because OSE and RTW-SE are two important predictors for various work outcomes and actual RTW after sickness absence [1, 22, 26]. This could include the assessment of working conditions to identify individuals with a higher risk for negative work outcomes and problems during RTW. Work-focused cognitive-behavioural interventions could further help employees receiving PT-W to acquire strategies to cope with high job demands and help them to work on negative perceptions of working conditions [9, 12]. Previous research found that work-focused (cognitive-behavioural) interventions increased RTW-SE and partly supported successful actual RTW among employees with CMD [9, 65, 66]. In addition, PT-W may offer opportunities for therapists to cooperate closely with occupational physicians, members of company integration management or other contact persons at the employee’s workplace [11, 37], which could support the adoption of necessary workplace adjustments based on the identification of unfavourable working conditions (e.g. agreements concerning work time, reduction of tasks). Our results thereby suggest that those interventions may especially focus on quantitative demands and development opportunities as well as social support for male and decision authority for female employees. Furthermore, longitudinal studies may help investigate causal and potential reciprocal relationships between working conditions and work-specific SE. Our results thereby strongly indicate that those studies should be accompanied by gender-sensitive analyses. Furthermore, experimental and qualitative research could help shed light on the mechanisms behind those relationships.

## Conclusions

Results suggest that high quantitative demands are associated with reduced work-specific SE, whereas psychosocial resources at work such as development opportunities, decision authority and social support are associated with better work-specific SE among employees receiving PT-W. Associations between decision authority, social support and work-specific SE were thereby shown to be moderated by gender. Those findings support usefulness of addressing potential reciprocal relationships between working conditions and work-specific SE during PT-W, but also leave room for further longitudinal analyses to investigate causal relationships between working conditions and work-specific SE in this study population.

## Supplementary Information

Below is the link to the electronic supplementary material.

Supplementary file1 (DOCX 29 KB)

## Data Availability

No datasets were generated or analysed during the current study.
