# Supplementary material for: Associations between Psychosocial Working Conditions and Work-Specific Self-Efficacy Beliefs Among Employees Receiving Psychotherapeutic Consultation at Work
Source: J Occup Rehabil. 2024 Nov 22;35(4):945–57. doi: 10.1007/s10926-024-10256-1 (PMC12575598; doi:10.1007/s10926-024-10256-1)
Supplement: Supplementary file 1 — Supplementary file1 (DOCX 29 KB) [file 10926_2024_10256_MOESM1_ESM.docx]

**Online Resource 1.** Graphical depiction of interaction trends


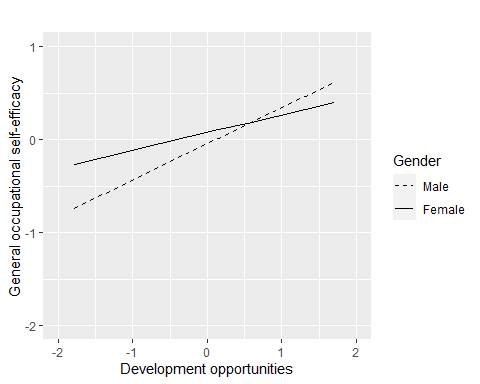


Figure S1 Interaction trend between development opportunities and gender on general occupational self-efficacy


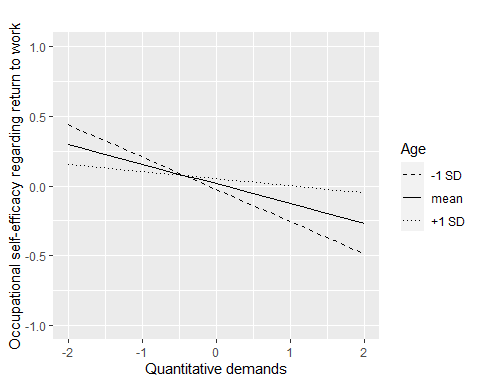


Figure S2 Interaction trend between quantitative demands and age on occupational self-efficacy regarding return to work


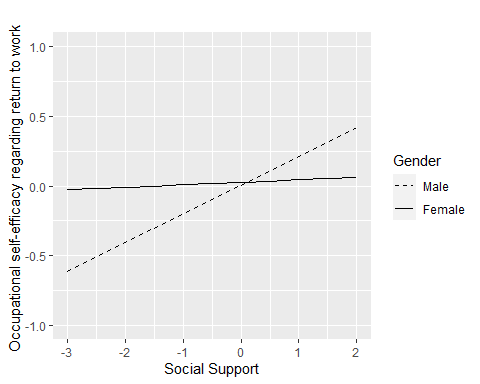


Figure S3 Interaction trend between quantitative demands and gender on occupational self-efficacy regarding return to work

**Article Title:** Associations between psychosocial working conditions and work-specific self-efficacy beliefs among employees receiving psychotherapeutic consultation at work

**Journal:** Journal of Occupational Rehabilitation

**Authors & Affiliations:** Jeannette Weber^1^, Marieke Hansmann^2^, Meike Heming^1^, Regina Herold^3^, Yesim Erim^3^, Nicole Hander^4^, Eva Rothermund^4^, Nadine Mulfinger^5^, Christoph Kröger^2^, Manuel Feißt^6^, Jolanda Brezinski,^6^ Fiona Kohl^1^, Peter Angerer^1^

^1^ Institute of Occupational, Social and Environmental Medicine, Centre for Health and Society, Medical Faculty and University Hospital Düsseldorf, Heinrich Heine University Düsseldorf, Düsseldorf, Germany

^2^ Institute of Psychology, University of Hildesheim, Hildesheim, Germany

^3^ Department of Psychosomatic Medicine and Psychotherapy, University Hospital of Erlangen, Friedrich-Alexander University Erlangen-Nürnberg (FAU), Erlangen, Germany

^4^ Department of Psychosomatic Medicine and Psychotherapy, Ulm University Medical Center, Ulm, Germany

^5^ Department of Psychiatry II, Ulm University and BKH Günzburg, Günzburg, Germany

^6^ Institute of Medical Biometry, University of Heidelberg, Heidelberg, Germany

**Corresponding author:** Jeannette Weber, Jeannette.Weber@uni-duesseldorf.de
